# Variability and forensic efficiency of 12 X-STR markers in Namibian populations

**DOI:** 10.1007/s00414-024-03299-9

**Published:** 2024-08-06

**Authors:** Luca Calò, Fabiano Gentile, Elisa Baio, Caterina Raschellà, Cristian Capelli, Alberto Marino

**Affiliations:** 1 Reparto Carabinieri Investigazioni Scientifiche di Parma - Sezione Biologia, Parma, Italy; 2 Department of Chemistry, Life Sciences and Environmental Sustainability, University of Parma, Parma, Italy; 3 Department of Medicine and Surgery, University of Parma, Parma, Italy

**Keywords:** X-STRs, Population database, Argus X-12 kit, African ethnic groups, Forensic parameters

## Abstract

STR loci localized on the X chromosome provide information additional to the autosomal markers routinely analyzed in forensic genetics, integrating genetic systems as Y-STRs and mitochondrial DNA in the investigation of complex kinship scenarios and mass disaster cases.

In this study we genotyped 12 X-STR loci in 251 male samples from four populations of Namibia in southern Africa using the Investigator Argus X-12 kit (Qiagen, Hilden, Germany). Forensic efficiency parameters indicated high power of discrimination in the considered populations. As part of our investigation, we highlighted partial linkage associations between loci within known linkage groups (LGs) and identified several occurrences of previously unreported out-of-ladder (OL) alleles.

Genetic distances between the Namibian populations here investigated and other African (Eritrea, Ethiopia, Somalia, Guinea, Cape Verde) and non-African (Germany, China, Philippines) populations using loci grouped in LGs mirrored their biogeographical distribution differently for each linkage group. Haplotype sharing within each LG revealed a high degree of population-specific types, hinting to the potential of these markers for ancestry applications.

These results highlight the importance to produce specific and freely available population databases especially for multi-ethnic countries. This novel dataset is expected to be of interest for population studies that need an accessible reference dataset of African regions not currently well represented, as well as possible relevance for forensic applications focusing on the biogeographic origin of samples.

**Supplementary Information:**

The online version contains supplementary material available at 10.1007/s00414-024-03299-9.

## Introduction

The analysis of X-linked STR markers has proven useful in solving kinship cases involving females and incest, as well as for identification purposes when data on reference parents is missing [1–4].

Several X-linked STRs have been identified, organized into four linkage groups with different degrees of linkage between markers [5]. Among these markers, the forensic community has selected a subset, which has been extensively characterized in terms of population variation and forensic informativity [6]. These markers have been also tested for their molecular efficiency when assembled in multiplex reactions [6].

One of the amplification kit used for the analysis of X-linked STRs is the Investigator®Argus X-12 (Qiagen, Hilden, Germany) that allows the simultaneous amplification of 12 STR loci. Although population studies about X chromosome polymorphisms are widespread in the literature, data on haplotype frequencies is not extensively available [7]. Moreover, an X-STRs open-access database is not present, beside the one originally developed by Szibor et al. [5] which contains only four populations to date (German, Ghanaian, Japanese and Chinese). Finally, as often the case for genetic studies, African populations have been only minimally investigated so far [7].

In order to tackle these issues, we genotyped a set of X-STRs in a group of population from Namibia in southern Africa, a region of the world particularly lacking data on X chromosome STRs [7]. In doing so, we characterized the degree of forensic informativeness of these markers, reported some cases of dropout alleles and extend the database on known off-ladder alleles. We also evaluate the relevance for these markers for investigations focusing on the biogeographic origin of samples.

## Materials and methods

### Samples collection and genotyping

Samples analysed in this work were collected in Namibia, whose population counts to about 2 700 000 inhabitants, living in an area of 823 145 km^2^ [8]. Namibia is a multi-ethnic country with 11 ethnic groups reported in the census, the majority belonging to communities speaking Bantu languages [8]. The collection of the samples was approved by the Oxford Tropical Research Ethics Committee (OxTREC; OxTREC 49–09 and OxTREC 42–11) [9–11]. The analyses involved 251 DNA samples collected from healthy male subjects living in Namibia, provided by the Department of Chemistry, Life Sciences and Environmental Sustainability, University of Parma, Parma, Italy. Collected samples belonged to individuals that self-identified as belonging to the following groups (number of analysed samples): *Mbukushu* (or *Hambukushu;* *n* = 59) and *Ovambo* (*n* = 82), two Bantu-speaking populations and *Xun* (*n* = 41) and *Khwe* (*n* = 69), two KhoeSan-speaking populations. The anonymity of the samples was ensured by the use of alphanumeric codes and coded DNA samples were stored in the laboratory. The focus on male samples simplified the phasing of the X chromosome genotypes and the recovery of haplotypes. At the point of sampling participants were asked to confirm that, to the best of their knowledge, they were not related to people already sampled in the same location/sampling session.

The Oragene® kit was used to collect samples and the genetic material was extracted following kit manufacturer’s instructions [12]. Samples were quantified with the Quantifiler™ Trio DNA Quantification kit, plate was loaded into the 7500 Real-Time PCR thermal cycler and the results analyzed using the HID Real-Time PCR Analysis software [13, 14]. The Investigator® Argus X-12 kit (Qiagen, Hilden, Germany) was used to amplify the following X-linked loci (Linkage Group): DXS10148, DXS10135, DXS8378 (LG1); DXS7132, DXS10079, DXS10074 (LG2); DXS10103, HPRTB, DXS10101 (LG3); DXS10146, DXS10134, DXS7423 (LG4). DNA amplifications were performed following the kit manufacturer’s recommended protocols [15]. Finally, PCR products were separated and detected on an ABI Genetic Analyzer 3500 xL using POP-4 polymer; alleles were called and binned by GeneMapper ID-X v1.4 software [14, 16].

### Data analysis

Intra- and inter-populations genetic diversity of the X-STR markers was estimated considering loci singularly or as part of one of the four LGs, as haplotypes. Alleles and haplotypes frequencies were calculated by counting alleles and haplotypes and dividing by the total number of samples analyzed. StatsX v2.0 software was used to calculate the following forensic efficiency parameters for loci considered singularly and in LGs: X chromosome haplotype diversity (HD), X-STRs markers’ power of discrimination (PD), polymorphism information content (PIC) and the mean exclusion chance (MEC) [17]. Pairwise testing of Linkage Disequilibrium (LD) (significance threshold: 0.05) and genetic distances parameters to other populations were estimated with the Arlequin v3.5.2 software [18]. The degree of LD between loci was measured within populations, to avoid the impact of the specific evolutionary history of each population on others. Slatkin’s Fst was estimated as measure of genetic distance between populations, using haplotype frequencies. Distances were calculated between the set of Namibian populations here investigated for the first time and eight additional populations from Europe, Asia and Africa, available in the literature (see Table 1). Distances between populations were calculated for each of the four X-STRs linkage groups and graphically represented through Neighbour-Joining (NJ) trees generated using Mega X v. 11.0.13 software [19]. All calculations were performed using the default settings of the programs.

**Table 1:** Reference populations for the genetic distance estimation based on Fst value

Geographic area	Country	Ethnic group	Bibliographic reference
East Africa	Eritrea	Afar	Bini et al. 2021
		Bilen	
		Hedareb	
		Kunama	
		Nara	
		Saho	
		Tigre	
		Tigrinya	
	Ethiopia	Tigray	Haddish et al. 2022
	Somalia	-	Tomas et al. 2012
West Africa	Cape Verde	-	Alfonso Costa et al. 2014
	Guinea Bissau	Baiote	Gomes et al. 2017
		Felupe	
		Papel	
		Bijago	
		Mancanha	
		BalantaMane	
		Mansonca	
		Cassanga	
		Nalu	
		Brame	
		Balanta	
		Mandinga	
		Beafada	
		Manjaco	
		Fula	
		FulaForro	
		FulaFula	
		Sussu	
Central Europe	Germany	-	Edelmann et al. 2012
Asia	China	Manchu	Xing et al. 2019
South-East Asia	Philippines	-	Salvador et al. 2018

Haplotype sharing between the considered populations (Namibian and others) was explored, to evaluate the potential informativity of X haplotypes in identifying the biogeographic origin of an individual for investigative purposes. The haplotype sharing function present in Arlequin v3.5.2 software was used for this purpose.

## Results

### Alleles/haplotypes frequencies, out of ladder and bi-allelic patterns

**Fig. 1:**
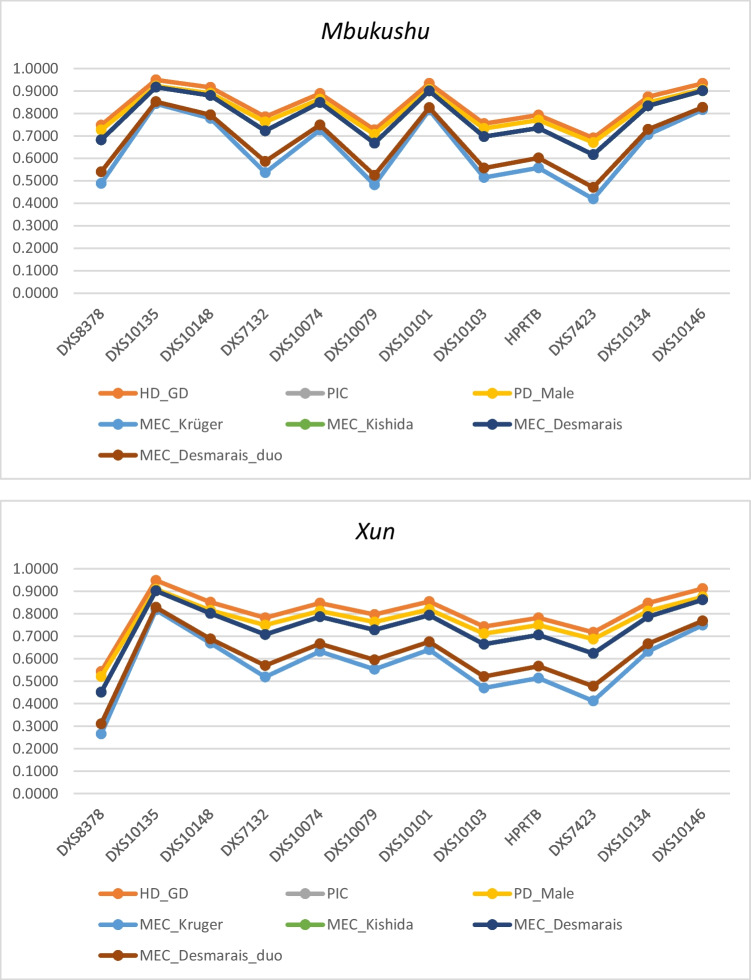

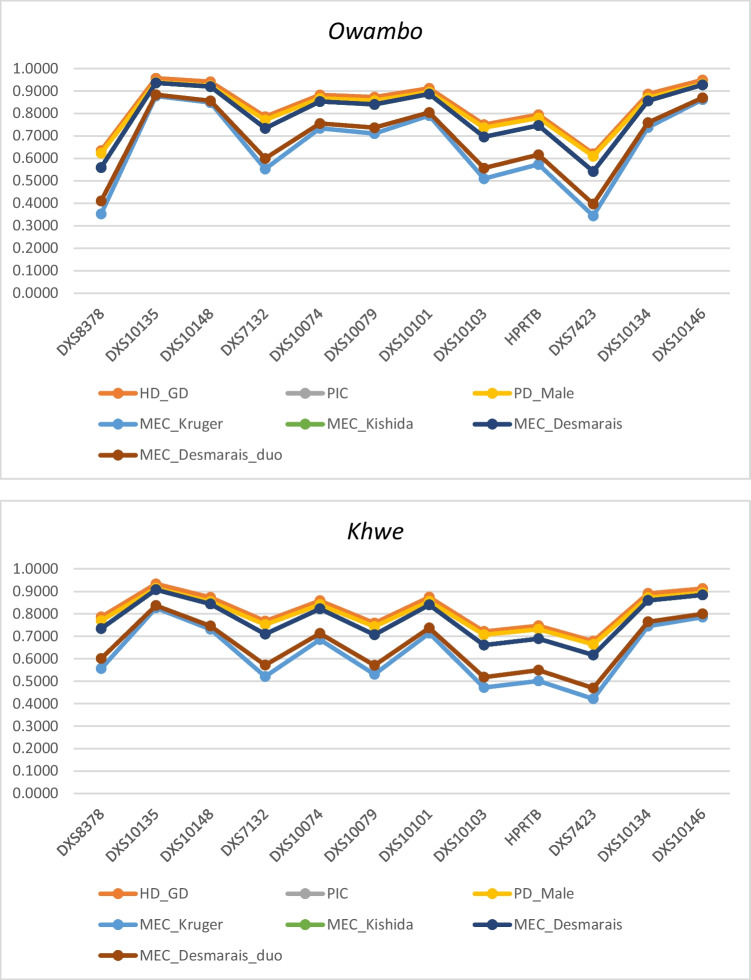
Forensic efficiency parameters relative to the 12 X-STR markers for each Namibian population

Genotyping of the 251 samples resulted in 242 complete profiles (50 *Mbukushu*, 41 *Xun*, 69 *Khwe* and 82 *Ovambo*). The missing 9 samples did not provide any amplification after multiple attempts and were not included. Several samples showed at least one out of ladder allele (OL), defined as any allele not included in the reference allelic set provided by the kit manufacturer. Some of these OL had been previously reported [20]. The newly identified ones are listed in Table 2. OL allele assignation was performed in accordance with their molecular weight. Some of the OL alleles were present in more than one population: the allele 8.1 at locus DXS7423 occurred in both the *Mbukushu* and the *Xun* and the alleles 28.3 and 29.3 at locus DXS10135 were shared between *Xun* and *Khwe*. None of the three newly identified alleles present in the *Ovambo* was shared with any of the other three populations. The two KhoeSan speaking populations (*Xun* and *Khwe*) are the ones where most of these newly identified alleles were detected, (5 unknown OL out of 11 total unknown OL, in both) (Table 2). Nine bi-allelic genotypes were observed at seven loci, two presenting the same alleles at locus DXS10101 in the *Xun* and two with different alleles at the same locus in different populations *(DXS10101, Xun and Ovambo)* (Table 3). The full set of allele frequencies in the four populations are reported in supplementary material (intermediate alleles with an incomplete repeat are reported without highlighting the incomplete allele, e.g. allele 13.3 was presented as 133) (Fig. 1-SM).

**Table 2:** Newly reported Out of Ladder (OL) alleles and their occurrence (number of individuals bearing the allele) within each population

Mbukushu	Xun			Khwe				Owambo	
DXS7423	DXS10135	DXS10146	DXS7423	DXS10135	DXS10148	DXS7132	HPRTB	DXS10079	DXS10134
8.1 (1)	28.3 (2)	33.1 (3)	8.1 (1)	28.3 (4)	22.3 (2)	14.1 (1)	9.1 (1)	17.1 (1)	27.1 (1)
	29.3 (1)			29.3 (3)				18.2 (1)	
	37.1 (3)

**Table 3:** Bi-allelic patterns and their occurrence (number of individuals bearing the allele) within each population

Mbukushu		Xun	Khwe				Owambo
DXS10134	DXS10148	DXS10101	DXS10103	DXS8378	DXS10074	HPRTB	DXS10101
35.3–36 (1)	20–20.1 (1)	30–33 (2)	19–21 (1)	10–11 (1)	15–17 (1)	9.1–13 (1)	32–33 (1)

Descriptive parameters concerning haplotype frequencies for each of the LGs are provided in Table 4. LG1 has therefore the highest potential to generate both different alleles and haplotypes. Only for the *Mbukushu* population, LG1 identifies a smaller number of haplotypes than LG4.

**Table 4:** Total number (N) of haplotypes, unique haplotypes and most common haplotype frequency for each linkage group (LG1, LG2, LG3 and LG4)

Mbukushu (N samples = 50)				Xun (N samples = 41)			
	N haplotypes	N single haplotypes	Most commo haplotype frequency		N haplotypes	N single haplotypes	Most commo haplotype frequency
LGI	42	35	0,06	LGI	32	26	0,10
LG2	42	34	0,04	LG2	26	15	0,07
LG3	41	35	0,10	LG3	31	23	0,10
LG4	46	42	0,04	LG4	30	20	0,07
Khwe (N samples = 69)				Owambo (N samples = 82)			
	N haplotypes	N single haplotypes	Most commo haplotype frequency		N haplotypes	N single haplotypes	Most commo haplotype frequency
LGI	42	29	0,06	LGI	70	61	0,05
LG2	37	19	0,09	LG2	66	53	0,04
LG3	42	29	0,10	LG3	57	57	0,07
LG4	43	27	0,07	LG4	70	70	0,04

### Forensic efficiency parameters

**Fig. 2:**
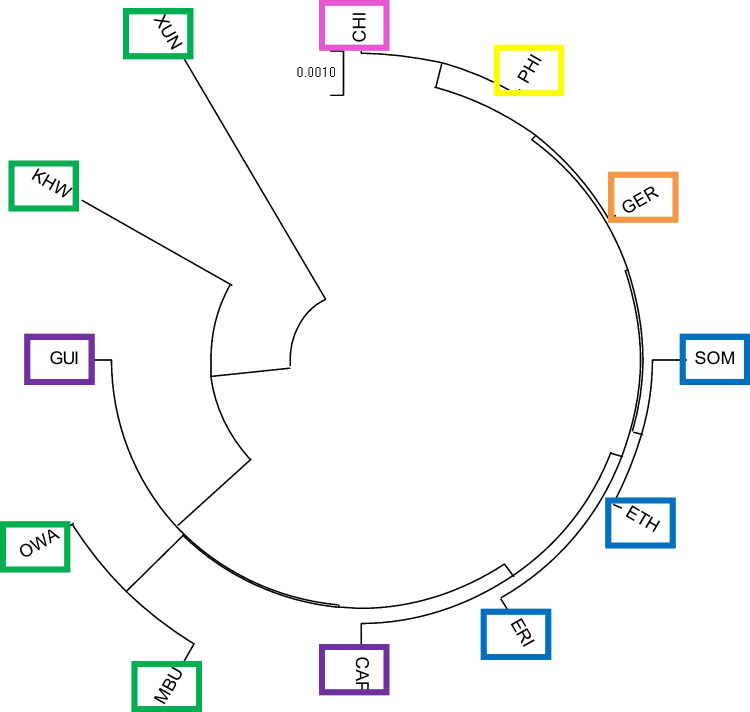
LG1’s Neighbour-Joining phylogenetic tree in circular pattern. The tree was elaborated using MegaX v. 11.0.13 software from the genetic distance matrix produced by Arlequin v3.5.2 software. The tree is drawn to scale, with branch lengths in the same units as those of the evolutionary distances used to infer the phylogenetic tree and the tree is root-free. Codes are boxed using different colours according to the following geographical macro-area: Southern Africa (MBU, XUN, KHW, OWA) – green; East Africa (ERI, ETH, SOM) – blue; West Africa (CAP, GUI) – purple; Europe (GER) – orange; Continental Asia (CHI) – pink; Southeast Asia (PHI) – yellow

Forensic efficiency parameters for the individual X-STR markers and for linkage group were separately evaluated using the StatsX software (Fig. 2 and Tabs. 1/2 -SM). No major differences are evident across populations (Fig. 1). Note that since the StatsX software deletes all incomplete profiles, parameters were computed on a total of *N* = 35 samples for the *Mbukushu* population, *N* = 24 for the *Xun* population, *N* = 48 samples for the *Khwe* population and *N* = 54 for the *Owambo* population.

### Linkage disequilibrium (LD)

Results of linkage disequilibrium tests are shown in supplementary material (Tab. 3-SM). Overall, the results confirmed the subdivision of the 12 loci into four linkage groups, with some pairs in each LG showing no significant association in the different populations. However, lack of significant association could be simply due to the small sample size analyzed in each population.

It is noteworthy the presence of LD between markers belonging to different LGs, unexpected by the physical localization of the makers on the X-chromosome. These observations were more common in the *Khwe* and *Owambo* populations and involved more often makers in LG1 and LG3.

### LG genetic distances and haplotype distribution

The four Namibian populations (*Mbukushu*-MBU, *Xun*-XUN, *Khwe*-KHW and *Owambo*-OWA) were compared to each other and to a set of worldwide reference populations (*N* = 8, Eritrea–ERI; Ethiopia–ETH; Somalia–SOM; Cape Verde–CAP; Guinea Bissau–GUI; Germany–GER; China–CHI; Philippines–PHI) using Slatkin linearized Fst. Distances were calculated using Arlequin v3.5.2 software for each linkage group, separately (Tab. 4-SM). Distance matrices were used to build Neighbour-Joining (NJ) trees with MegaX v. 11.0.13 software (Fig. 3-SM).

The pair of populations showing the largest distance in each LG were the *Mbukushu* and *Xun* for LG1 (Fst = 0.01441), *Khwe* and Eritrea for LG2 (Fst = 0.02047), *Khwe* and *Xun* for LG3 and LG4 (Fst = 0.01712 and 0.01660, respectively).

Of the four LGs, LG1 appears as the LG group showing the best fitting between geography and genetics. The NJ dendogram separates Africans and non-Africans (in accordance with the Out of Africa model for the origin of *Homo sapiens*), groups Eastern and Western-Southern Africans separately and places the two Asian groups closer to each other [21–24] (Fig. 2). Some of these patterns are also present in the trees based upon the other LGs, but never all together (Fig. 3-SM).

### Population specific haplotypes and haplotypes sharing

Considering the results based on the trees, we explored the degree of haplotype sharing across all the populations for the 4 LGs. Haplotypes distribution and patterns of shared haplotypes are listed in the supplementary material. Namibian populations analysis was carried out only considering complete haplotypes for each LG, excluding haplotypes in which one or more markers had a missing value. LG1 and LG4 generally presented a greater number of population-specific haplotype than LG2 and LG3. Both the percentage of the population-specific haplotypes (PSHh, estimated out of the total number of haplotypes for each population) and the percentage of individuals presenting a specific haplotype (PSHi, estimated out of the total number of individuals for a given population) were calculated. The population with the greater number of specific haplotypes was Germany (for all LGs) while the one that presented a lower number was *Xun* population for both LG1 and LG3, *Khwe* population for LG2, *Mbukushu* and Philippines populations for the LG4. As expected the number of novel haplotype increases with the number of tested individuals, until a plateu is reached when large datasets are tested. Our results confirmed the presence of a clear correlation between the number of haplotypes and the number of different individuals (Fig. 4-SM). Interestingly, although the relatively smaller sample size of the Namibian populations compared to the reference dataset, PSHh and PSHi values in these populations were similar to those in the reference populations (values were lower than 15% for both LG2 and LG3 and between 20–40% for LG4, see “%Hapl” in the Supplementary Material). LG1 values differed the most between Namibia populations and the others: values ranged within 40–60% in Namibian groups while were below 40% in the reference populations (except for Guinea).

## Discussion

### X chromosome drop-outs, multi-allelic loci and out of ladder alleles in Namibian populations

The analysis of X-STR markers using the Investigator Argus X-12 kit (Qiagen, Hilden, Germany) in four Nambian populations resulted in several cases of allele drop-out (DO), in markers DXS10148, DXS10101, DXS10146, DXS10135, DXS7132 and DXS10079. Drop-outs can occur when nucleotide variants are present in the primers binding sites or when samples present DNA degradation [7, 25–29]. However, the low degradation index estimated for the samples showing DO events estimated through the ratio small/large autosomal probes using Quantifiler™ Trio DNA Quantification kit suggests a variation in the primer binding region as the most plausible explanation for the observed DO events.

Bi-allelic patterns were observed for across populations at different loci: the DXS10134 and DXS10148 about *Mbukushu* samples, DXS10101 about both *Xun* and *Owambo* populations, DXS10103, DXS8378, DXS10079 and HPRTB markers for the *Khwe* population. Bi-allelic patterns could be the result of amplification or typing process artifacts or else they could represent a mosaicism condition. Bi- and tri-allelic patterns in the X-STR loci were already been described in the literature [29–33].

Several out-of-ladder (OL) alleles were detected, not uncommon phenomenon when using the Investigator Argus X-12 [28, 30–32]. A subset of these were observed here for the first time (Table 2).

### Forensic efficiency

The most polymorphic and informative marker for all the four Namibian population was DXS10135 (PIC *Mbukushu* = 0.9172 with 21 several alleles, PIC *Khwe* = 0.9076 with 23 several alleles, PIC *Xun* = 0.9027 with 20 several alleles, PIC *Owambo* = 0.9363 with 25 several alleles) while the less informative and polymorphic marker was the DXS7423 in the *Mbukushu* (PIC = 0.6165 with 5 alleles), *Khwe* (PIC = 0.6169 with 5 alleles) and *Owambo* (PIC = 0.5422 with 5 alleles) and DXS8378 marker in the *Xun* population (PIC = 0.4510 with 6 several alleles). These observations are in accordance with data in the literature [25, 34–36].

There were no major differences between parameters estimated across the four linkage groups, all very close to the maximum value of 1. Overall, the obtained results confirmed the forensic informativeness of the 12 X-STR markers in the studied populations.

### Population genetics analysis

The Linkage Disequilibrium tests supported the assemblage in four linkage groups of the 12 X-STR markers, with some observations of lack of linkage within LGs and presence of linkage across LGs (Tab. 3-SM). Population sub-structure, absence of random mating and genetic drift are all possible evolutionary scenarios explaining these discrepancies [37, 38]. On the other hand, these observations could be the result of stochastic effects due to limited size of our samples. Notable, the presence of significant LD between markers DXS10135 (LG1) and DXS7423 (LG4) localized at the X-chromosome opposite ends (Xp22.31 and Xq28 positions, respectively) has been already reported [36]. However, it is worth mentioning that, despite early observations [5, 39], recombination events between associated markers and incomplete independence between markers belonging to different LG have been extensively reported [36, 40–43].

Across the phylogenetic trees built using genetic distances between haplotypes for each LG, the one referring to LG1 data was the one that was closest to the real biogeographic distribution of the considered populations. In fact, African and Non-African populations were associated to two different branches, the two Asian populations close to each other (PHI and CHI) and African populations were further subdivided into Southern Africa (MBU, OWA, KHW, XUN), Eastern Africa (ERI, ETH, SOM) and Western Africa (CAP and GUI).

About LG1 tree, Eastern Africa populations were phylogenetically close together as well as two of the study populations (*Owambo* and *Mbukushu*). On the other hand, in the LG2 tree we noted a populations subdivision in a cluster that showed a different distribution compared to the real one: a single group included Germany and *Xun* while *Khwe* and *Mbukushu* were phylogenetically quite far, such as Eritrea, than the others. This could be the effect of a genetic drift that involved these ethnic groups.

Concerning to the LG3 tree, two of the Southern Africa populations (OWA and XUN) formed a single cluster thus highlighting their phylogenetic closeness unlike the *Khwe* population, which was slightly distant from these and close to the *Mbukushu*. In the same tree, we noted some clusters clearly not steady with the populations geographical distribution such as the German/Ethiopia/Eritrea phylogenetic association.

Finally, in the LG4 tree East Africa populations (ERI, ETH and SOM) were phylogenetically close together as well as those belonging to West Africa (CAP and GUI). Moreover, *Owambo* population (Southern Africa) appeared phylogenetically close to the West Africa populations. On the contrary, *Xun* and *Khwe* populations (Southern Africa) were far from each other and also from all the others showing two separate clusters due to a genetic drift effect that involved them, probably [37].

In all cases the Asian populations (CHI and PHI) were placed within the same cluster.

Therefore, populations genetic non-homogeneity emerged both by the results and our considerations, probably due to the high intra-population inbreeding levels: hence, the need and the importance to generate population-specific databases [25, 28, 44, 45].

### Haplotype sharing and biogeographic origin identification

The study of X-STR markers is known to be a useful tool for identifying the geographical origin of a biological sample donor [7, 25, 39]. In a forensic scene, this is very important especially in forensic cases where any additional information could be crucial in the characterization of the origin of the biological material. The X-typing of the 251 male samples and the comparison of the genotypes with reference databases allowed us to identify a list of population-specific haplotypes for each considered population. In general, LG1 was the one with a greater number of population specific haplotypes (PSHi range: 60–19% PSHh range: 69–22%) followed by the LG4 > LG2 > LG3. Overall LG based haplotypes appear to have the potential for application related to the determination of the geographical origin of individuals whose origin are unknown, but further analyses specifically testing for the degree of bio-geographical association of LG haplotypes are necessary for their routine application in the forensic context.

## Conclusion

The analysis of the 12 STRs loci in the four Namibian populations confirmed the forensic informativeness of these markers. The identification of several drops out, OL alleles and biallelic loci confirms the need to extend the survey of genetic variation to other populations beyond Europe. Our work extends the set of population data from Africa, with a particular relevance for Southern Africa, a geographic region with still very limited X-STR data. We are aware that the population sample analyzed is relatively small. However, being one of the few investigations of X-STRs in populations from Southern Africa, we believe that it represents a significant contribution to the general goal of the forensic community of implementing representative reference databases of all human populations. Given that an updated X-STR database is not yet available, it is highly desirable to implement one, either by developing it from scratch or by extending STR repositories already available (i.e. NIST STRBase or STRidER).

## Supplementary Information

Below is the link to the electronic supplementary material.

Supplementary file1 (XLSX 1473 KB)

## Data Availability

All data generated or analysed during this study are included in this published article [and its supplementary information files].
